# Supplementary material for: Spatial proteomics of Onchocerca volvulus with pleomorphic neoplasms shows local and systemic dysregulation of protein expression
Source: bioRxiv. 2024 Oct 17:2024.10.15.618383. Preprint. [Version 1] doi: 10.1101/2024.10.15.618383 (PMC11507698; doi:10.1101/2024.10.15.618383)
Supplement: 1 [file NIHPP2024.10.15.618383v1-supplement-1.pdf]

bioRxiv preprint doi: https://doi.org/10.1101/2024.10.15.618383; this version posted October 17, 2024. The copyright holder for this preprint (which was not certified by peer review) is the author/funder, who has granted bioRxiv a license to display the preprint in perpetuity. It is made available under aCC-BY 4.0 International license.

Supporting information

Table S1. Excel spreadsheet with the functional annotation, peptides, spectra count and NSAF values of the *Onchocerca volvulus* parasite-related-proteins and classification of the matched proteins by BLASTP searches against *Homo sapiens* and *O. volvulus* databases.

Table S2. Excel spreadsheet with the functional annotation, peptides, spectra count and NSAF values of the *Onchocerca volvulus* parasite-related-proteins and classification of the matched proteins by BLASTP searches against Wolbachia database.

Table S3. Primers for OVOC8391 amplification. Primers were designed for one of the low similarity with other parasites regions.

Table S4. Complete list of enriched gene ontology pathways, InterPro and KEGG domains. *O. volvulus*-derived proteins detected in the polymorphic neoplasm tissue, only proteins that were supported by at least 2 unique peptides in two biological replicates were used. Each tab represents one complete analysis.

S1 Fig. Overview of H&E stained *O. volvulus* sections. Worm sections which were used for LCM and which material was dissected using an image which had been manually color-coded to help keeping track which tissue should be dissected with the laser. A is a pleomorphic neoplasm worm and C is a healthy female. B and D are examples of images used at the LCM. Ut= uterus, b= bodywall, neo= neoplasm, m= male mo=morulae, Orange= neoplasm or embryos, green= gut; yellow= body wall, periwinkle= uterus wall.

S2 Fig. Alignment for with filarial orthologues. The sequence for OVOC8391 and its filarial orthologues were retrieved from WormBase Parasite. MegAlign Pro from DNA Star was used to align the sequences using OVOC8391 as reference.
